# Safety, Efficacy, and Ease of Insertion of Gnana Laryngeal Airway (GLA-4): A Prospective Clinical Study Utilizing the Unique Laryngeal Mask Airway With a Suction Tubing

**DOI:** 10.7759/cureus.49735

**Published:** 2023-11-30

**Authors:** Shahab Ahmadzadeh, Naina Singh, Matthew J Sharpe, Hirni Patel, Gabriel Lavespere, Noah J Spillers, Giustino Varrassi, Steven J Alexander, Sahar Shekoohi, Elyse M Cornett, Alan D Kaye

**Affiliations:** 1 Department of Anesthesiology, Louisiana State University Health Sciences Center, Shreveport, USA; 2 School of Medicine, Louisiana State University Health Sciences Center, Shreveport, USA; 3 Department of Pain Medicine, Paolo Procacci Foundation, Rome, ITA; 4 Department of Physiology, Louisiana State University Health Sciences Center, Shreveport, USA

**Keywords:** airway management, laryngospasm, oropharyngeal secretions, periglottic seal, intubation, lma, laryngeal mask airway

## Abstract

Introduction: Utilizing laryngeal mask airways to maintain patients' airways is advantageous because it enables the anesthesiologist to keep the patient spontaneously inhaling and is less traumatic to the airway than intubation. Newer designs such as the Gnana laryngeal mask airway design permit real-time suctioning while the mask is on a patient.

Methods: This is a prospective observational study of the efficacy of Gnana laryngeal airway 4 (GLA-4) in 50 patients undergoing colonoscopy. Induction and maintenance of anesthesia were provided with propofol; GLA-4 was applied to secure the airway; and correct placement was verified.

Results: Fifty patients were included in the study (44% female, 56% male, mean age: 56.5 years, mean BMI: 33.3). Twelve patients were assigned American Society of Anesthesiologists (ASA) class 2, and 38 were assigned ASA class 3. The first attempt of GLA-4 insertion was successful in 47 patients, and two attempts were required for the successful placement of the GLA-4 in two patients. The successful placement was not achieved in one patient. The average time to successful insertion was 27.1 ± 3.9s. The average volume of oropharyngeal secretions suctioned through the suction catheter was 9.96 ± 2.31 mL. No intraoperative or postoperative complications occurred in the 50 patients. There were no reports of sore throat, hoarseness, dysphagia, or cough immediately postop.

Conclusion: GLA-4 can be inserted safely with adequate periglottic occlusion. This laryngeal mask is unique and desirable due to its ability to evacuate oropharyngeal secretions while in place to prevent laryngospasm. To establish the role of GLA-4 in broader clinical situations, additional clinical trials and studies are required.

## Introduction

Supraglottic airway (SGA) devices have transformed the approach to routine and emergency airways in pediatric and adult patients since the advent of laryngeal mask airways (LMAs) in the late twentieth century [1]. Although still considered a novel approach, supraglottic airway devices are utilized globally in a wide range of settings and surgical procedures. Utilizing LMAs to maintain patients’ airways is advantageous as it allows the anesthesiologist to either keep the patient spontaneously breathing or place the patient on a ventilator to maintain the airway [2]. As LMAs are versatile, they can be used as airway rescue devices, serving as conduits for intubation. For example, in urgent cases where establishing the airway is time-sensitive for various reasons, a laryngeal mask airway can quickly and safely secure the airway through which an endotracheal tube can be placed to further secure the airway [3-5]. Apart from emergent and elective surgical cases, LMAs have been successful in out-of-hospital settings [6]. Notably, intubating laryngeal mask airway versus bag-mask ventilation resulted in higher 24-hour survival rates in out-of-hospital cardiac arrest patients.

Various types of laryngeal mask airway devices are available, and each model has a slightly different feature, making each of them advantageous for particular reasons. Presently, the versions of supraglottic devices are the LMA-Classic, LMA-Unique, LMA-Fastrach, LMA-Proseal, LMA-Supreme, LMA-Ambu, and LMA-SoftSeal. Recently, a second-generation supraglottic airway device, the Gnana laryngeal airway 4 (GLA-4) was created by an Australian company (Gnana Medical Australia Pvt. Limited, Gnana Medical, Australia), and its application in clinical practice was outlined in the *Turkish Journal of Anesthesiology and Reanimation* [7]. The GLA-4 is advantageous in design as it has an additional suction port on the convex side to suck the oral secretions. To determine the utility of the novel second-generation GLA-4, we conducted a study at Louisiana State University Health Science Center at Shreveport that evaluated its success in terms of time to insertion, ease of insertion, and the number of required attempts in obese adult patients undergoing elective surgeries.

## Materials and methods

This investigation was conducted at Louisiana State University Health Science Center at Shreveport, a level 1 trauma center located in Shreveport, Louisiana, and included patients undergoing elective colonoscopy. This study was approved by the Institutional Review Board of Louisiana State University Health Sciences Center in Shreveport (approval number: 00001755). The study design was a prospective observational study. The primary outcome was ease of insertion of GLA-4 as measured by first pass success rate. Secondary outcome measures were the number of attempts, the prevalence of successful insertion, the time taken for successful insertion, and complications of GLA-4, if any.

All participants undergoing the research study had signed an informed consent regarding the use of a supraglottic airway device as a potential modality of establishing the airway during their respective surgical procedures. The efficacy of GLA-4 was studied in a total of 50 patients. All patients were classified according to the American Society of Anesthesiologists (ASA) physical status and allocated into either ASA 2 or ASA 3 categories. Preoperative airway evaluation included opening of the mouth more than 2.5 cm, normal temporomandibular joint mobility, neck movement, and a modified Mallampati score to rule out difficult airways.

The patients' ages ranged from 25 to 82 years old. Upon arrival in the operation theater, an intravenous (IV) line was placed. Standard monitoring included the continuous electrocardiogram (ECG), heart rate (HR), non-invasive blood pressure (NIBP), oxygen saturation (SpO_2_), end-tidal carbon dioxide (EtCO_2_), and respiratory mechanics parameters (compliance, resistance, measured minute ventilation, and peak airway pressures). Once successful induction was achieved, the GLA-4 was applied to secure the airway according to the manufacturer’s guidelines. Before using the GLA-4, the laryngeal airway mask cuff for inflation and deflation was checked. The back of the mask was lubricated with a water-soluble lubricant before placing it in the larynx.

The GLA-4 was advanced to the posterior pharyngeal wall with the index finger. The mask was then inflated until it moved slightly out of the hypopharynx. The time between picking up the GLA device and obtaining an effective airway was recorded. Correct placement of the GLA-4 was verified by auscultating for equal and bilateral breath sounds over both lung fields, absence of breath sounds over the epigastric region, visualization of EtCO_2_ on the monitor, and absence of auscultatory air leak around the GLA-4. The number of insertion attempts was recorded. A maximum of three attempts of device placement were permitted per patient. The size 4 of the second-generation GLA-4 used for the first attempt was based on the patient’s weight according to the manufacturer’s instructions. If the device did not function effectively, the following manipulations were performed in sequence: The insertion depth was increased; the device was rotated, and the device was withdrawn slightly. If these maneuvers failed to achieve an effective airway, the device was removed after two attempts, and another second-generation laryngeal mask was inserted. The ventilator was adjusted to achieve effective oxygenation and ventilation using pressure support mode. The aim was to achieve SpO_2_ of ≥95% and EtCO_2_ of 35-40 mmHg using an inspired oxygen fraction (FiO_2_) ≤ 0.5 with a respiratory rate of 10-16 min-1 and tidal volume of 8-12 ml/kg. Maintenance of anesthesia was achieved with propofol infusion. After completion of the procedure, the airway device was removed when the patient was awake and resumed spontaneous breathing.

Although the salivary secretion sucked out by the GLA-4 was not the primary outcome variable in this study, this was nonetheless observed as an additional variable. This was done by the oropharyngeal secretions drawn in the syringe from the suction port of the GLA-4. Postoperatively, patients were assessed for sore throat, hoarseness, dysphonia, cough, or any other adverse effects just before discharge from the postoperative care area.

## Results

After evaluating patients for eligibility criteria, 50 were included in the study. About 44% of participants were female, and 56% were male undergoing elective colonoscopy. Out of 50 patients, 12 were assigned ASA class 2, and 38 were assigned ASA class 3. The patient population had a mean age of 56.5 years and a mean BMI of 33.3 kg/m^2^. All patients had a Mallampati score of I-III.

**Figure 1:**
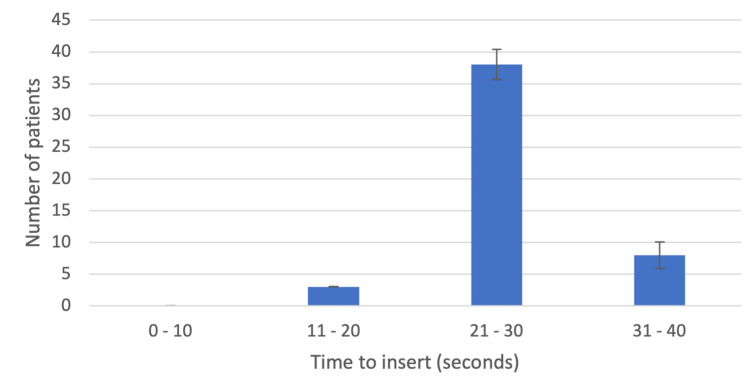
A bar graph demonstrating the time (in seconds) taken to insert the GLA-4 in the 49 patients who had a successful placement. Placement took between 21 and 30 seconds for a majority of these patients. GLA-4: Gnana laryngeal airway 4.

The colonoscopy procedure duration for the cases ranged from as short as 15 minutes to as long as 62 minutes. Of the 50 patients, 49 underwent induction of general anesthesia with propofol, and one received etomidate. The first attempt to insert the GLA-4 was successful in 47 patients, and two attempts were required for the successful placement of the GLA-4 in two patients. Correct placement was verified in 49 patients. One patient did not have a successful placement. Time to insertion was assessed by starting a timer on the monitor, and the average time to successful insertion was 27.1 ± 3.9 s (Figure 1).

**Figure 2:**
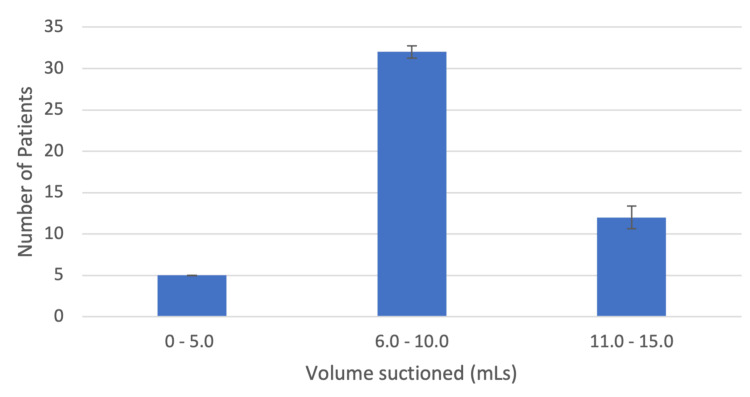
A bar graph demonstrating the amount of secretions suctioned for this group of 49 patients in which GLA-4 placement was successful. About 6-10 mL of secretions were suctioned for 65% of the patients. GLA-4: Gnana laryngeal airway 4.

Oropharyngeal secretions were measured in terms of total volume suctioned once the GLA-4 was secured and throughout the surgical case until the removal of the GLA-4. The average volume of oropharyngeal secretions suctioned was 9.96 ± 2.31 mL (Figure 2). There were no intraoperative and postoperative complications in the 50 patients who underwent GLA-4 application for airway management. After the surgery, patients were evaluated in the post-anesthesia care unit (PACU) where they were asked if symptoms of sore throat, hoarseness, dysphagia, or cough were present to assess for postoperative complications of using GLA-4. None of the 50 patients reported positive symptoms of sore throat, hoarseness, dysphagia, or cough immediately postop.

## Discussion

Compared to endotracheal intubation, supraglottic airways offer several advantages and limitations. Tracheal intubation has been associated with a higher risk of sore throat in the postoperative period compared to SGAs [1,8-10]. Additional advantages of SGA use include the reduced incidence of laryngospasm on emergence and reversible bronchospasm [11]. SGAs play a vital role in the ASA practice guidelines for difficult airway management [12-16]. Although contraindicated in situations involving elevated risk of regurgitation, SGA use is still appropriate in emergent airway scenarios [17]. The original purpose of a gastric port, as first seen in the LMA-ProSeal, was to aid in recognizing SGA malpositioning [18]. The GLA-4 suction tube makes it possible to suction oropharyngeal secretions when the laryngeal mask stays in the oropharyngeal cavity. This is important because secretions can cause laryngospasm [19]. This ability of GLA-4 provides significant benefits over other types of LMAs, especially in patients with medical conditions such as postnasal drip and sinusitis [20]. Innovations in SGA design have resulted in their use in patient populations and procedures previously regarded as problematic, such as laparoscopic procedures and obese patients [21-25]. A finding noted with SGA use in such scenarios was an increase in leak fraction, particularly during abdominal insufflation [26]. A recent cross-sectional observational study revealed increased airway events with SGA use in obese patients [27]. Still, it did not display a significant decrease in airway events with tracheal intubation in this population [27].

Further investigation should be taken into the performance of the GLA-4 with regard to air-leak fraction and its effect on adequate ventilation. Although the use of SGAs is widespread, it does not come without its disadvantages and complications. Placement of an SGA can lead to microscopic or macroscopic trauma, and microscopic trauma is rarely associated with more serious sequelae. Major trauma includes damage to vital structures such as the tongue, uvula, epiglottis, and laryngeal apparatus. Nerve injuries have previously been described as a result of SGA placement. Injuries to the lingual nerve, hypoglossal nerve, and laryngeal nerves have all been associated with SGAs [28-30]. A study by Marjot has suggested that increased pressures within an SGA cuff can exceed capillary perfusion pressure in the hypopharynx [31]. Other more commonly known disadvantages and complications include aspiration risks, loss of airway on insertion or during maintenance, and displacement after insertion [7].

There are a few limitations to this research study. High-risk patients, such as patients with expected difficult airways, cardiorespiratory or cerebrovascular disorder, class III obesity, and pregnant women, were excluded from the study. Therefore, the results and conclusions cannot be extrapolated in these high-risk populations. Moreover, the sample size of 50 may be seen as insufficient; however, our main focus was on the ease of insertion and other physical characteristics of GLA-4.

## Conclusions

None of the patients developed any complications. GLA-4 can be safely inserted with a satisfactory periglottic seal. The benefit of suctioning the oropharyngeal secretions while the laryngeal mask is in situ makes this laryngeal mask unique. Further clinical studies as broader trials are required to establish the role of GLA-4 in wider clinical situations.
